# Survey of HBsAg-positive pregnant women and their infants regarding measures to prevent maternal-infantile transmission

**DOI:** 10.1186/1471-2334-10-26

**Published:** 2010-02-15

**Authors:** Yan Guo, Jianqiong Liu, Liping Meng, Hu Meina, Yukai Du

**Affiliations:** 1 The Department of Maternal and Child Health, School of Public Health, Tongji Medical College, Huazhong University of Science and Technology, 430030, China

## Abstract

**Background:**

Intrauterine infection is the main contributor to maternal-infantile transmission of HBV. This is a retrospective study of 158 HBsAg-positive pregnant women who delivered children from Jan 1st, 2004 to Dec.31th, 2006 in Wuhan City, China. We investigated the measures taken to prevent maternal-infantile transmission of hepatitis B virus and the infection status of children.

**Methods:**

HBsAg-positive pregnant women were selected by a random sampling method when they accepted prenatal care in district-level Maternal and Child Health Hospitals. On a voluntary basis, these women completed questionnaires by face-to-face or phone interviews. The collected data were used to evaluate the immunization programs that pregnant women had received for preventing hepatitis B maternal-infantile transmission.

**Results:**

Among the 158 women, 143(90.5%) received Hepatitis B immune globulin during pregnancy, and 86.0% of their children were given Hepatitis B immune globulin and Hepatitis B vaccine. The rate of cesarean section was 82.3%, and 28.5% of these were aimed at preventing HBV infection. The rate of bottle feeding was 51.9%, and 89.0% of bottle feeding cases were for the purpose of preventing HBV infection. There were 71 cases of participants who were HBeAg-positive. Compared with the HBsAg+ HBeAg- group (only HBsAg-positive), the HBsAg + HBeAg+ group (HBsAg-positive and HBeAg-positive) had significantly higher rates of the caesarean section and bottle feeding resulting from hepatitis B (*P *< 0.05). Five cases were HBsAg-positive by Umbilical Cord Blood detection. The intrauterine infection rate of newborns was 6.7%. The chronic HBV rate of children was 4.0%.

**Conclusion:**

Most HBsAg positive pregnant women have a growing awareness of maternal-infantile transmission of Hepatitis B virus and are receiving some form of preventative treatment, like combined immunization. Caesarean and bottle feeding are very common, often primarily to prevent transmission. Relatively few intrauterine infections were identified in this sample, but many infants did not appear to seroconvert after vaccination.

## Background

Hepatitis B virus infection is a serious public health problem. China has a high incidence of HBV infection, with a 10% HBsAg positive rate in the general population. There are more than 130 million chronic carriers of HBV in China, 30% ~50% of which can be attributed to maternal-infantile transmission[1]. Intrauterine infection is the main contributor to maternal-infantile transmission of HBV. The intrauterine infection rate of HBsAg -positive pregnant women is 5%~40.1% [2,3]. The existing combined immunization program, whereby high-risk newborns receive Hepatitis B immune globulin (HBIG) and hepatitis B vaccine, can effectively decrease mother-to-child transmission of HBV during delivery and postpartum, but the immune failure rate is still high, at 20% ~30%[4].

Using HBIG before childbirth[5] has been shown to be effective in preventing intrauterine HBV infection, but the efficacy of any specific program of immunization is as yet inconclusive. There are very few retrospective studies on the implementation status and outcomes of combined immunization programs since they were first put into practice. In the present study, we retrieved all immunization and treatment records for HBsAg positive pregnant women and their infants from Jan 1st, 2004 through Dec.31th, 2006 in the city of Wuhan. Combined with a designed questionnaire, we investigated the immunization program that pregnant women received to prevent maternal-infantile transmission of hepatitis B and also evaluated the status of HBV infection in the children after combined immunization.

## Methods

### Subjects

The HBsAg-positive pregnant women and their children were selected as study subjects from among those who had accepted prenatal care in the district-level Maternal and Child Health Hospitals of Wuhan City, between Jan 1st, 2004 to Dec.31th, 2006. Of these, 158 cases were brought into the study. Based on a hepatitis laboratory tests, they were divided into two groups: the HBsAg+ HBeAg- group (only HBsAg-positive) and the HBsAg + HBeAg+ group (HBsAg-positive and HBeAg-positive). Those who accepted combined immunization and had umbilical cord blood test results and the vein test results after the full vaccination were selected for interview, and the data were used to evaluate the status of their children's HBV infection after combined immunization.

### IRB Approval/Informed consent

The study protocol will be reviewed and approved by the Institutional Review Board of Tongji Medical College, Huazhong University of Science & Technology, 2007(02). Data files and completed questionnaires are kept under lock and key to ensure confidentiality of respondents.

Informed consent was obtained from the HBsAg-positive pregnant women and their children.

### Data collection

Adopting the retrospective method, correlative information was retrieved from maternal prenatal health care manuals of the HBsAg-positive pregnant women. This included factors such as check-ups during early pregnancy, treatments to prevent maternal-infantile transmission of HBV, method of delivery and feeding etc. Trained investigators conducted face-to-face or telephone interviews with the women based on a voluntary and confidential principle using uniform questionnaires. The content of the questionnaires included the profiles and test results of HBV, while also determining the immunization and HBIG treatment before and after delivery, delivery situation and feeding patterns etc. Questionnaires were checked after being collected, and the entry-related information was analyzed by being contrasted with recorded information from manuals.

### Diagnostic Criteria

Diagnostic criteria for pregnant women infected with HBV were in accordance with the diagnosis criteria of the National Conference on Viral Hepatitis in October 2000 in Xi'an, China [6]. The diagnostic criterion for intrauterine infection of newborns was HBV-DNA >1.0e+003 in umbilical cord blood test results (PCR) during delivery. The diagnostic criterion for chronic HBV infection of children was HBsAg-positive serum for six months or more[7,8].

Combined Immunization consisted of injection of HBsAg positive pregnant women with HBIG during pregnancy, and their children were later given HBIG and Hepatitis B vaccine.

### Statistical analysis

EpiData 3.02 was used to establish the database. The data were converted into an SAS database after being checked by being input twice, and then analyzed statistically with SAS version 8.1 software. Descriptive analysis (the calculation of average or frequency, proportion or rate) was conducted. Single-factor analysis: (χ^2 ^test or t test) was used to compare the difference of qualitative data or quantitative data, respectively.

## Results

### HBsAg positive description

A total of 158 cases were HBsAg positive in the random sample (2026 pregnant women were enrolled), corresponding to a HBV positive rate of 7.8 percent. Among the 158 cases investigated, 127 cases (80.4%) had only HBsAg-positive (HBsAg+ HBeAg- group) and 31 cases (19.6%) had the combined HBsAg and HBeAg-positive (double - positive group). The statistical analysis showed no significant differences in age, occupation, education level, income, or other general socio-economic status between the HBsAg+ HBeAg- group (only HBsAg-positive) and HBsAg + HBeAg+ group (HBsAg-positive and HBeAg-positive), (*P *> 0.05), indicating that the two groups were comparable.

### Immunization program

Among the 158 cases of HBsAg positive pregnant women, 143 (90.5%) cases received HBIG, and 86.0% of their children were given HBIG and Hepatitis B vaccine. Among the 158 women, 82.3% accepted cesarean section, of which 28.5% were aimed at preventing HBV infection. The rate of bottle feeding was 51.9%, and 89.0% of bottle feeding cases were for the purpose of preventing HBV infection. In contrast, in HBsAg negative pregnant women (1868/2026), the cesarean section ratio was 73.47%, and bottle feeding ratio was 41.80%. Both of these were significantly lower than the rates of HBsAg positive pregnant women (χ^2 ^= 5.9250, *P *= 0.0149; χ^2 ^= 6.0647, *P *= 0.0138).

There was no significant difference in the percentage of women who received HBIG during pregnancy between the HBsAg+ HBeAg- group and the HBsAg + HBeAg+ group (Table 1). However, the caesarean section rate of the HBsAg + HBeAg+ group was significantly higher than the HBsAg+ HBeAg- group (*P *< 0.05). More double-positive than single-positive women opted for bottle feeding (*P *< 0.05).

**Table 1:** Comparison of intervention measures between the HBsAg+ HBeAg- group and HBsAg + HBeAg + group

	Single-positive group (127)	Double-positive group (31)		
				
Interruption measures	n (%)	n (%)	χ^2^	*P*
Pregnancy HBIG Injected			0.9726	0.3240
yes	113 (89.0)	30 (96.8)		
no	14 (11.0)	1 (3.2)		
Infant immunization			1.2889	0.2563
Hepatitis B vaccine	23 (18.1)	3 (9.7)		
Hepatitis B vaccine & HBIG	104 (81.9)	28 (90.3)		
Combined immunization			3.4802	0.0621
yes	95 (74.8)	28 (90.3)		
no	32 (25.2)	3 (9.7)		
Cesarean section			1.7115	0.1908
yes	102(80.3)	28(90.3)		
no	25(19.7)	3(9.7)		
Cesarean section due to HBV infection			18.2321	<0.0001
yes	20(19.6)	17(60.7)		
no	82(80.4)	11(39.3)		
Bottle feeding			26.7994	<0.0001
yes	53(41.7)	29(93.6)		
no	74(58.3)	2(5.4)		
Bottle feeding due to HBV infection			3.9302	0.0474
yes	44(83.0)	29(100.0)		
no	9(17.0)	0(0.0)		

### HBV infection of children

To determine the HBV status of children after combined immunization, 75 cases were chosen from the investigated HBsAg-positive pregnant women. Umbilical cord blood and vein blood from these women and their children were tested for HBV serological markers by lab tests after full vaccination. Of these 75 cases, 54 were from HBsAg+ HBeAg- group and 21 were from HBsAg + HBeAg+ group. In total, 5 cases were HBsAg-positive as determined by umbilical cord blood test (1 from HBsAg+ HBeAg- group, and 4 from HBsAg + HBeAg+ group). The overall intrauterine infection rate was 6.7%. A total of 20 out of 75 cases were HBsAb positive, for a positive rate of 26.7%.

Intrauterine infection rate of the HBsAg + HBeAg+ group (19.1%) was significantly higher than that of the HBsAg+ HBeAg- group (1.9%), emendation χ^2 ^= 4.6875, *P *= 0.0304 (Table 2). In all, 3 cases that were HBsAg-positive at birth turned negative after the full vaccination. Their mothers all were HBsAg and HBeAg positive. The rate of chronic HBV infection in children was 4.0%.

**Table 2:** Comparison of the HBV infection of children between the HBsAg+ HBeAg- group and HBsAg + HBeAg+ group

	Single-positive group (127)	Double-positive group (31)		
				
Infants Outcome	n (%)	n (%)	χ^2^	*P*
Intrauterine infection			4.6875	0.0304
yes	1(1.9)	4(19.1)		
no	53(98.1)	17(80.9)		
HBsAb positive			0.0541	0.8161
yes	14(25.9)	6(28.6)		
no	40(74.1)	15(71.4)		
HBV serological markers full-negative			3.5465	0.0597
yes	39(62.2)	11(52.3)		
no	13(37.8)	10(47.7)		

(HBV serological markers include the five items in table 1.)

## Discussion

The HBV carrying rate of pregnant women in Wuhan has reportedly decreased in recent years[9]. The present survey showed that from 2004 to 2006, the HBV carrying rate was 7.8% for this region. This rate was not only higher than that of developed countries, such as European countries and the United States[10], but it was also higher than that of many developing countries [11,12]. This might be attributed, at least in part, to the increase in HBV screening.

Maternal-infantile transmission is an important transmission route for HBV; thus, strategies aimed at interrupting this pathway would largely reduce the number of new infections and would mitigate the suffering imposed by the disease on the families, society, and individuals. In the current study, maternal-infantile transmission was low with combined immunization, in which the pregnant women receive HBIG injection during pregnancy and their newborns get HBIG and Hepatitis B vaccine after birth. This is in agreement with another study, which suggested that up to 95% of maternal-infantile transmission can be blocked by using mother-and-baby combined immunization [13]. In our survey, 86% of the HBsAg-positive pregnant women received HBIG before delivery, and their children were given HBIG and Hepatitis B vaccine. The percentage of chronic HBV infection in infants with the combined immunization program was 4.0%, which was much lower than those without vaccination (80%~95%)[14], and also lower than those whose mothers had not received HBIG (10%~15%)[15]. It is possible that a HBsAg-positive reaction could change to negative in children with combined immunization[16]. Despite this, most children still lack HBsAb and are extremely susceptible to HBV infection. The reason could be two possibilities. One was insufficient or inefficient vaccination, the other was the times of producing antibodies differing in diverse people, which leads to different outcomes after the full-course vaccination. If children have no HBsAb after full vaccination, they will be advised to be revaccinated for HBV.

Mothers with positive HBeAg are at high risk for intrauterine transmission. Therefore, women who are positive for HBeAg should be taken into special consideration to prevent maternal-infantile transmission of HBV. When a mother is HBV positive, a caesarean section may be done to protect the baby from direct contact with her blood and other bodily fluids. This survey has shown that more than 80% of pregnant women with positive HBsAg opted for cesarean section. Some studies suggest that cesarean section could reduce the likelihood of HBV infections in the infants [18,19], whereas other researchers found no difference in maternal-infantile transmission of HBV between women with cesarean section and natural birth. This is an important consideration, as cesarean section will increase medical costs, prolong length of stay in hospital, and can add additional complications due to operation syndrome.

More than half of the investigated mothers also opted for bottle feeding. As seen with cesarean section, double- positive pregnant women were more worried about the risk of transmission of HBV via breast milk, which is still controversial [20,21].

Although recent studies have shown the effectiveness of a combined immunization program in prevention of vertical HBV transmission and the benefits of active and passive immunization of infants have been recognized, the effects of injecting HBIG before delivery are still controversial [22,23]. At present, almost all HBsAg-positive pregnant women and even HBsAg-positive, HBeAb-positive and HBcAb-positive women, are not recommended for treatment with HBIG during pregnancy at most hospitals in Wuhan City. Thus, the increase in intrauterine infection and chronic infection is still a concern. According to the principles of evidence-based medicine, a well-designed and forward-looking study is needed, with large samples in multi-center organizations. We can then make a comprehensive assessment of the effects of giving HBIG to pregnant women for prevention of maternal-infantile transmission of HBV.

## Conclusion

Most HBV positive pregnant women have a growing awareness of maternal-infantile transmission of Hepatitis B virus and are receiving some form of preventative treatment. For example, their children are given HBIG and Hepatitis B vaccine and/or HBsAg positive pregnant women are injected with 3-5 doses of HBIG during pregnancy. Caesarean section and bottle feeding are very common, often performed primarily to prevent HBV transmission. Relatively few intrauterine infections were identified in this sample, but many infants did not appear to seroconvert after vaccination.

## Competing interests

The authors declare that they have no competing interests.

## Authors' contributions

YG carried out the retrospective studies, participated in the design of the study and performed the statistical analysis and drafted the manuscript. JL, LM and MH carried out the field investigation and participated in the statistical analysis. YD conceived of the study, and participated in its design and coordination and helped to draft the manuscript. All authors read and approved the final manuscript.

## Pre-publication history

The pre-publication history for this paper can be accessed here:

http://www.biomedcentral.com/1471-2334/10/26/prepub
